# Epigenetic Modulation of Stem Cells in Neurodevelopment: The Role of Methylation and Acetylation

**DOI:** 10.3389/fncel.2017.00023

**Published:** 2017-02-07

**Authors:** Martyna Podobinska, Ilona Szablowska-Gadomska, Justyna Augustyniak, Ioanna Sandvig, Axel Sandvig, Leonora Buzanska

**Affiliations:** ^1^Stem Cell Bioengineering Unit, Mossakowski Medical Research Centre, Polish Academy of SciencesWarsaw, Poland; ^2^Pharmacology Department, Institute of Mother and ChildWarsaw, Poland; ^3^Department of Neuromedicine and Movement Science, Faculty of Medicine and Health Sciences, Norwegian University of Science and Technology (NTNU)Trondheim, Norway

**Keywords:** epigenetics, methylation, acetylation, stem cells, neurodevelopment, neuroplasticity

## Abstract

The coordinated development of the nervous system requires fidelity in the expression of specific genes determining the different neural cell phenotypes. Stem cell fate decisions during neurodevelopment are strictly correlated with their epigenetic status. The epigenetic regulatory processes, such as DNA methylation and histone modifications discussed in this review article, may impact both neural stem cell (NSC) self-renewal and differentiation and thus play an important role in neurodevelopment. At the same time, stem cell decisions regarding fate commitment and differentiation are highly dependent on the temporospatial expression of specific genes contingent on the developmental stage of the nervous system. An interplay between the above, as well as basic cell processes, such as transcription regulation, DNA replication, cell cycle regulation and DNA repair therefore determine the accuracy and function of neuronal connections. This may significantly impact embryonic health and development as well as cognitive processes such as neuroplasticity and memory formation later in the adult.

## Introduction

The development of the nervous system is regulated by a multitude of intracellular molecular and cellular signals interacting with the extracellular microenvironment in a temporal and spatial manner. These signals induce expression of genes involved in lineage commitment, differentiation, maturation, migration and cell survival. In addition, silencing of genes responsible for the maintenance of stem cells in a pluripotent state and for cell fate decisions are important hallmarks of neurodevelopment (Lilja et al., 2012). Neural stem cells (NSCs) are able to proliferate and self-renew and also give rise to all neural lineage cells in the central nervous system (CNS): neurons, astrocytes and oligodendrocytes (Altman, 1962; Gage, 2000).

After development is completed, NSCs are still present in the adult brain throughout its entire lifetime in two neurogenic niches: the subventricular zone (SVZ) lining the lateral ventricles, and the subgranular zone (SGZ), which is part of the dentate gyrus (DG) of the hippocampus (Eriksson et al., 1998). It is well documented in pioneering (Hsieh and Gage, 2004) and more recent studies that epigenetic alteration plays a crucial role in the maintenance of the resident NSC multipotent state as well as in their differentiation process (Mohamed Ariff et al., 2012).

The term “epigenetics” was coined by Conrad Waddington (1905–1975) in 1942 to describe interactions between genotype and the environment, which together shape the expressed characteristic traits of an organism, defined as the phenotype (Waddington, 1942). Different definitions of the term epigenetics exist and a clear consensus is still lacking. The name epi- (Greek: *ɛπí*- over, above, outer)-genetics refers to exogenous dynamic mechanisms which change gene expression without changing the DNA sequence. Epigenetic changes can however be inherited through subsequent cell divisions. Each cell type has a unique gene expression profile (transcriptome) and also a distinctive chromatin signature (Sha and Boyer, 2009). Interestingly, epigenetic changes are dependent on environmental factors. It has been documented that maternal behavior during pregnancy or prenatal stress may lead to epigenetic alterations in the offspring (Wu et al., 2004; Morgan and Bale, 2011).

Epigenetic mechanisms that control changes in gene expression levels can be divided into three main groups: (i) DNA methylation; (ii) histone and chromatin modifications, including histone variants; and (iii) non-coding RNAs (ncRNAs) such as long non-coding RNA (lncRNA) and small non-coding RNAs (sncRNAs), including microRNAs (miRNAs); and small interfering RNAs (siRNAs; Jenuwein and Allis, 2001; Sun et al., 2008; Mohamed Ariff et al., 2012; Fenoglio et al., 2013).

**Figure 1:**
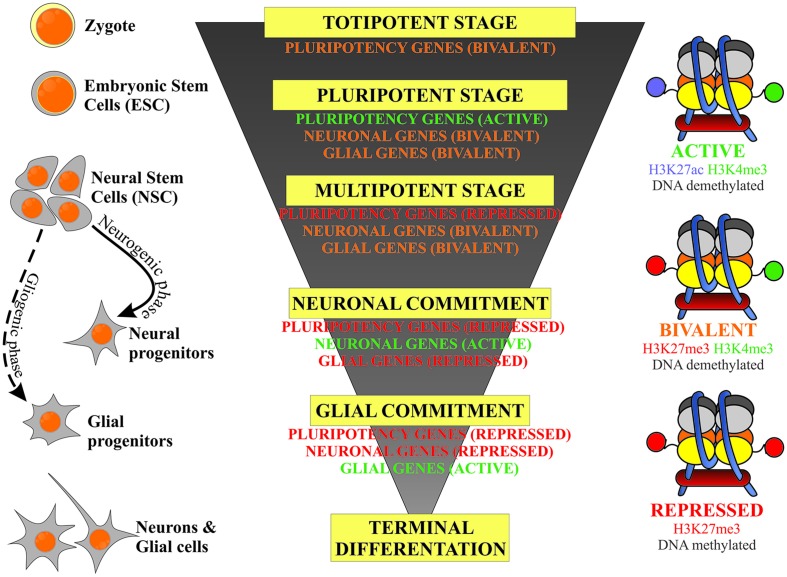
**Schematic representation of the correlation of the stem cells’ developmental hierarchy with their epigenetic status based on the balance between inhibitory (DNA and H3K27 methylation) and permissive (H3K4 methylation) epigenetic marks**.

This review article will focus on the regulation of DNA methylation and two types of histone modification: methylation and acetylation and their relevance to stem cell fate decisions in neurodevelopmental processes. The epigenetic events are considered to act in a switch-like mode (Hoffmann et al., 2015), however the potential of the cell to undertake developmental decisions, stemness/lineage commitment and further differentiation is highly dependent on the activity of the genes typical for the defined stage of development. A schematic correlation of neurodevelopmental hierarchy of stem cells, along with their epigenetic status is presented on Figure 1. Developmental genes are referred to as being at an activated (on), repressed (off) or bivalent (poised) stage. This is linked to the pattern of DNA and histone permissive and inhibitory epigenetic marks, which are further discussed in this review article.

## DNA Methylation and Its Relevance to Stem Cells and Neurodevelopment

### DNA Methylation

DNA methylation is a crucial process in embryogenesis and normal development and one of the most frequently investigated epigenetic modifications. It is involved in regulation of gene expression (Keshet et al., 1986), X-chromosome inactivation (Heard et al., 1997), genomic imprinting (Li et al., 1993; Denomme and Mann, 2013), regulation of chromatin structure (Bernstein et al., 2007), transposon silencing (Walsh et al., 1998) and control of telomere length (Gonzalo et al., 2006). Methylation of cytosine at the C5 position is catalyzed by various DNA methyltransferases (DNMTs), which catalyze the transfer of a methyl group (-CH_3_), derived from the S-adenosylmethionine (SAM; methyl donor) onto cytosine and, as a result, lead to formation of 5-methylcytosine (5mC; Cheng et al., 1993). There is also an alternative form of DNA methylation; Kriaucionis and Heintz (2009) discovered 5-hydroxymethylcytosine (5hmC), an unusual DNA nucleotide that occurs frequently in the mouse brain.

The DNA methylation pattern is established during embryonic development by *de novo* DNMTs (DNMT3A and DNMT3B supported by DNMT3L), while maintenance of this pattern is linked with activity of the DNMT1 (Razin and Szyf, 1984; Li and Zhao, 2008). Lister et al. (2009) suggest that DNMT3A may be responsible for catalyzing cytosine methylation at non-5′-C-phosphate-G-3′(CpG) sites.

An interesting fact is that in mammals the DNA of mature sperm and egg is highly methylated compared with somatic cells. However, the largest extent of this methylation disappears during the preimplantation developmental stage (genome wide demethylation). After implantation, the embryo undergoes a *de novo* DNA methylation process (Reik et al., 2001; Denomme and Mann, 2013). Furthermore, *Dnmt1* or *Dnmt3a* and *Dnmt3b* knockout mice die during mid-gestation (Li et al., 1992; Okano et al., 1999; Ueda et al., 2006). There are two mechanisms for the regulation of gene expression by DNA methylation: (i) DNA methylation prevents the binding of certain transcription factors, which blocks transcription; and (ii) methylated CpG dinucleotides are recognized by a family of proteins containing methyl-CpG-binding domain (MBD) such as MBD1 and methyl CpG binding protein 2 (MeCP2), which actively block their binding sites and are consequently responsible for gene inactivation (Li and Zhao, 2008; Sun et al., 2008). MBD-proteins can block transcription alone or in cooperation with enzymes responsible for histone modification (Nan et al., 1998). Interestingly, recent studies have shown that actively transcribed genes have high levels of gene body methylation (Lister et al., 2009; Wu et al., 2010).

### Regulation of DNA Methylation

Traditionally DNA methylation was considered as stable, irreversible epigenetic modification. However, studies conducted in recent years revealed methylation changes in postnatal brains (Guo et al., 2011a).

It has been proposed that the demethylation process may take place in two different ways; either active or passive. The passive way is associated with suppression of DNMT1, while active demethylation occurs enzymatically and is catalyzed by TET1—tet methylcytosine dioxygenase 1 (5mC hydroxylase) (Wu and Zhang, 2010; Guo et al., 2011b) involved in oxidative reaction of 5mC to 5hmC transition.

The other mechanism, which is involved in active DNA demethylation is associated with growth arrest and DNA-damage-inducible alpha (GADD45A) protein. GADD45 proteins are involved in many processes associated with cellular response to stress. They have been implicated in cell-cycle control, DNA repair and suppression of cell growth (Kaufmann et al., 2011). High GADD45A expression is associated with global DNA demethylation (Barreto et al., 2007). *Gadd45* genes mediate the repair-based DNA demethylation and may occur by three mechanisms as described (Teperek-Tkacz et al., 2011; Niehrs and Schäfer, 2012). Some authors have suggested that DNA methylation patterns may be useful epigenetic markers in characterizing different types of pluripotent and differentiated cells (Watanabe et al., 2012). The basis for their assumptions is the fact that there are different patterns of DNA methylation in pluripotent stem cells, during their differentiation and in terminally differentiated cells. It has been demonstrated that the DNA methylation profile undergoes dynamic changes during the process of cell differentiation, particularly in HCP promoters (promoters with high CpG content; Meissner et al., 2008).

### The Role of DNA Methylation in the Neural Commitment and Differentiation of Stem Cells

*Dnmt1, Dnmt3a* and *Dnmt3b* are expressed during CNS development and differentiation (Feng et al., 2007; Hutnick et al., 2009). As indicated above, DNA methylation has been shown to play an important role during embryonic development but is also significant in the formation of the nervous system as well as for NSC differentiation (Stroud et al., 2011; Szulwach et al., 2011; Wu et al., 2011; Chen et al., 2013, 2014). Alterations in DNA methylation during CNS development are consistent with the regulation of chromatin-modifying enzymes occurring during embryonic development (Gapp et al., 2014). For example, DNMT1 and DNMT3 maintain DNA methylation and regulate the division of neural progenitor cells and mature neurons as well as play an important role in adult neurogenesis and synaptic plasticity of mature neurons. Furthermore, *Dnmt1* and *Dnmt3a* deletion in mouse forebrain excitatory neurons caused a decrease in DNA methylation, abnormal neuron size and dysregulation in expression of genes important for synaptic plasticity (class I MHC and STAT1). These changes resulted in dysfunction in long-term plasticity in the hippocampal CA1 region, and caused learning and memory deficits (Feng et al., 2010). It was also shown that lack of DNMT1 results in perturbation of resident populations of neural stem/precursor cells and post-mitotic neurons. Specifically, the differences were, respectively, in terms of reduced numbers of neurons and a reduction of cortical and hippocampal volumes, and a negative effect on neuronal survival (Noguchi et al., 2015). Moreover Noguchi et al. (2016) showed that presence of DNMT1 is essential for hippocampal DG development, given that its lack in early development leads to poor granule cell layer in the adult DG. It was also revealed that during neurogenesis DNMT1 plays a pivotal role in the regulation of neural differentiation. Such control of NSC differentiation also serves the purpose of preventing astrocytes from premature differentiation (Noguchi et al., 2016).

DNMT3A influences mouse NSC maturation and differentiation. During neurogenesis, DNMT3A is expressed in embryonic neural precursor cells (NPCs) within the SVZ and in postmitotic CNS neurons as well as in oligodendrocytes (Feng et al., 2005). In order to understand the role of DNMT3A in NSC differentiation and proliferation, Wu et al. (2012) used NSC derived from DNMT3A-deficient mouse embryonic stem cells (ESCs). These *Dnmt3a^−/−^* NSCs were globally hypomethylated and exhibited an increased proliferation rate compared to control NSCs, which indicates that DNMT3A plays an important role in mouse NSC proliferation. Moreover, the same authors observed altered timing of NSC differentiation. In early passages (P3), *Dnmt3a^−/−^* NSCs exhibited precocious differentiation towards both astrocytes and oligodendrocytes, while neuronal differentiation was not impaired. Furthermore, loss of DNMT3A led to an increased number of both astrocytes and oligodendrocytes (Wu et al., 2012).

During development, neural precursors in the SVZ give rise to neurons and, subsequently, astrocytes and oligodendrocytes. This developmental, hierarchical transition is closely associated with epigenetic remodeling of many genes. The principal role in astrogliogenesis is played by Glial Fibrillary Acidic Protein (GFAP) transcription, which is activated by developmental demethylation of its promotor. Since the process of GFAP activation is triggered by STAT3 transcription factor, the methylation of the CpG at the STAT3 binding site in the GFAP promotor represses gliogenesis (Takizawa et al., 2001; Feng et al., 2007; Namihira et al., 2009). These data focused attention to DNA methylation as a critical determinant of astrocyte differentiation in the fetal brain.

Recent studies have shown that during the perinatal period, the male and female mouse brains differ in DNMT enzyme activity in the highly sexually dimorphic preoptic area (POA). Exposure to gonadal steroids leads to a decrease in DNMT activity, which results in a reduction of DNA methylation (i.e., a reduction of fully methylated CpG sites) and activation of masculinizing genes. Moreover, DNMT3A inhibition caused brain masculinization, which resulted in male copulative behavior. These findings indicated that brain feminization is an active process maintained by higher levels DNA methylation (Nugent et al., 2015).

The family of *Gadd45* genes is another important player in CNS development. Candal et al. (2004) have shown that *Gadd45gamma* is involved in the development of medaka fish brain, specifically in cell fate decisions, by affecting cell cycle exit. Further research demonstrated that, during mouse neural development, mainly two of *Gadd45* genes, *Gadd45a and Gadd45g*, are expressed with slight temporospatial differences. High *Gadd45a* expression is present in the neural tube during its closure, in the dorsal root and cranial ganglia VII-X, and in the olfactory epithelium. Expression of *Gadd45g* is also observed in the neural tube, dorsal root and cranial ganglia VII-X, and olfactory epithelium, but also in neural precursors, forebrain, midbrain and hindbrain (Kaufmann et al., 2011).

Furthermore, recent studies have shown that *Gadd45b* expression (but not *Gadd45a*, or *Gadd45g*) and DNA methylation in the developing rodent amygdala are different in individuals of different sexes (Kigar et al., 2016).

Many studies have shown that epigenetic regulation affects activity-dependent mature brain functions. Activity-dependent neurogenesis in the adult hippocampus is one of the mechanisms involved in neural plasticity. However, the specific mechanisms of action of this phenomenon are poorly understood. Ma et al. (2009) discovered that *Gadd45b* is needed for activity-induced DNA demethylation of some of the gene promoters involved in adult neurogenesis. Region-specific DNA demethylation, performed by GADD45B, enabled expression of paracrine neurogenic niche factors (BDNF, FGF-1, FGF-2) in mature neurons, which induced activity-dependent adult neurogenesis (Ma et al., 2009).

Zhou and his group have shown that DNA methylation was programmed along with neural tube development by the pattern of appearance of DNA 5mC and 5hmC epigenetic modifications (Zhou, 2012). They further investigated differences between 5mC and 5hmC methylation sites in the brain (Chen et al., 2014). They discovered that 5hmC is located in alternate regions to 5mC, appears at different times during neurodevelopment and is connected with different MBD proteins. The spatiotemporal pattern of 5mC and 5hmC DNA methylation sites is closely associated with the state of differentiation of NSCs in the neural tube. Appearance of 5hmC is always preceded by the presence of 5mC. It was suggested that the surge of 5mC is connected with priming of NPCs for differentiation. Additionally, Stroud et al. (2011) have shown that the timing of 5hmC appearance is closely related with initiation of neuroepithelial cell differentiation. 5mC and 5hmC reveal different binding partners. While 5mC preferably binds to MBD1 and MeCP2, 5hmC is associated with MBD3. Moreover, negative correlation between the amount of 5hmC and MeCP2 levels is observed. 5mC is mostly distributed in the promoter regions of the genes and is co-localized with repressive histone marks, H3K9me3 and H3K27me3, while 5hmC is localized in the gene body, downstream from the transcription start site (TSS) and is co-localized with the H3K4me2 histone mark, which is a marker of active chromatin (Stroud et al., 2011). Szulwach et al. (2011) examined 5hmC occurrence in mouse cerebellum and hippocampus during neurodevelopment. They found a correlation in the spatial distribution between 5hmC and NeuN-positive neuronal cells in the granule cell layer in developing and adult cerebellum, wherein the amount of 5hmC increased in the latter. In calbindin-positive Purkinje cells the amount of 5hmC was increased in both developing and adult cerebellum, compared to NeuN-positive cells. However, the progenitor cells in the external granule layer, which are a highly proliferative fraction, lack 5hmC. In the hippocampus, these authors observed very low levels or lack of 5hmC in immature (NeuN-negative) neurons. The amount of 5hmC clearly increased in NeuN-positive cells in the DG granule cell layer in both developing and adult mice. Together, these data suggest that 5hmC clearly appears in mature neuronal cells, which implies a role in neuronal development (Szulwach et al., 2011). It is thus suggested that 5hmC is associated with euchromatin and plays an important role in maintaining the activity of genes characteristic for specific NSC subtypes (Stroud et al., 2011; Szulwach et al., 2011; Wu et al., 2011; Chen et al., 2014).

The most recent studies, using single cell immuno-identification and cell-specific quantitative methylation assays, revealed cell-wide DNA de-methylation and subsequent re-methylation of Purkinje Neurons in the developing cerebellum (Zhou et al., 2016). This process involved both 5mC and 5hmC. This is the first time when such global de-methylation has been observed beyond totipotent stages of development.

The correlation of different MBD proteins with the activated euchromatin and inactive heterochromatin sites (respectively MBD1 and MBD3) corresponds to an earlier observation by Zhao et al. (2003) that the absence of MBD1 causes reduced neuronal differentiation and increased genomic instability in NSCs and that adult *Mbd1^−/−^* mice exhibit deregulation in the DG of the hippocampus during adult neurogenesis.

TET (10 to 11 translocation) TET1, TET2 and TET3 proteins are dioxygenases that catalyze the conversion of the modified genomic base 5mC into 5hmC and are involved in further oxidative DNA demethylation process (Tahiliani et al., 2009). Thus, TET occupancy at gene promoters is negatively correlated with levels of DNA methylation.

The active participation of TET proteins in neural fate specification has been the subject of intense investigation given that in mammals it occurs abundantly in ESCs and neurons (Wu and Zhang, 2011; Li et al., 2014). Two different lines of evidence confirmed that non-promotor based 5hmC methylation sites are involved in maintaining active chromatin states of neurogenic genes (Wu et al., 2010; Zhang et al., 2013). It was first documented that DNMT3A dependent non-proximal promoter methylation triggers expression of neurogenic genes by functionally antagonizing Polycomb repression (Wu et al., 2010). In addition Zhang et al. (2013) have shown TET1 associated regulation of neural progenitor proliferation in the developing cortex as well as in a population of adult NSCs.

The precise and dynamic pattern of 5mC and 5hmC distribution within chromatin as the specific sites of DNA methylation located either in gene promoters or within the gene body also raise a more general question regarding the role of global methylation/demethylation and their link with the activity of the specific developmental genes. The mechanism underlying the widely accepted observation that DNA 5mC methylation at proximal promoters at CpG islands facilitates silencing of cell type–specific genes, while such lineage restriction is attenuated when 5hmC methylation occurs in non-proximal promoters at euchromatic DNA in of transcriptionally permissive (poised) developmental genes, remains to be elucidated.

## Histone Modifications and Their Relevance to Stem Cells and Neurodevelopment

**Figure 2:**
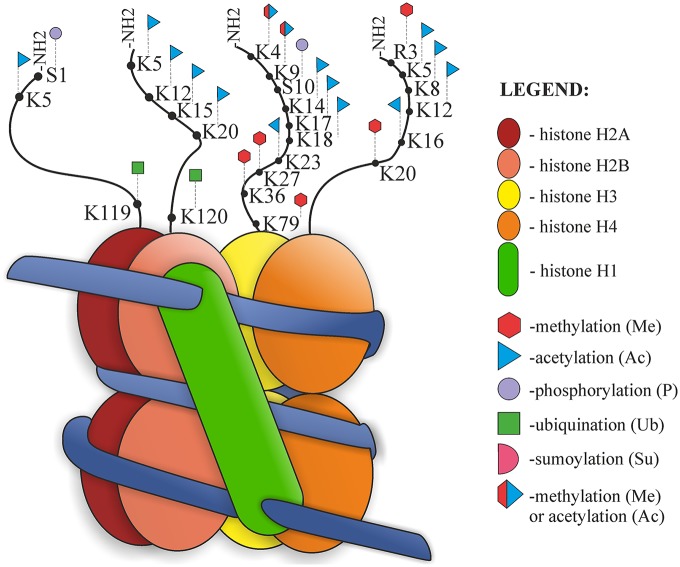
**Nucleosome organization and histone post-translational modifications (PTMs)**.

The structure of the nucleosome and its post-translational modifications (PTMs) are presented in Figure 2. Nucleosomes are the basic units of chromatin structure, consisting of DNA and histones. DNA with a length of 146 bp is wrapped in 1.67 left-handed superhelical turns around an octameric histone core which comprises a (H3–H4)_2_ tetramer and two H2A–H2B dimers. Nucleosomes are joined by 10–50-bp-long stretches of unwrapped linker DNA and linker histone (H1), which together are involved in chromatin compaction (Luger et al., 1997; Richmond and Davey, 2003).

Histones are small alkaline proteins consisting of a globular C-terminal domain and an N-terminal tail, which is positively charged and extends outwards from the nucleosome (Jenuwein and Allis, 2001). Histone tails are highly basic due to numerous lysine and arginine residues and play an important role in the structural stability of the nucleosome and in chromatin compaction. Regulation of the structure of nucleosomes is possible due to internucleosomal interactions between N-terminal tails and DNA or other parts of histones (Arya and Schlick, 2009). There are also sites of numerous PTMs, such as acetylation, phosphorylation, methylation, ubiquitination, SUMOylation, ADP-ribosylation, deimination, proline isomerization, crotonylation and citrullination. Both chromatin structure and histone modifications affect the availability of nuclear factors presented to the DNA. PTM can alter chromatin organization and regulate different processes, such as DNA replication, chromatin assembly after replication, transcription or DNA repair processes (Hanks et al., 1983; Shiio and Eisenman, 2003; Cuthbert et al., 2004; Nelson et al., 2006; Shogren-Knaak et al., 2006; Kouzarides, 2007; Ismail et al., 2010; Biswas et al., 2011; Martinez-Zamudio and Ha, 2012; Rothbart and Strahl, 2014). Jenuwein and Allis (2001) proposed the “Histone Code Hypothesis”, suggesting that different combinations of histone modifications may have different outcomes and can interact with each other in a synergistic or antagonistic way. According to this hypothesis, modification marks on the histone tails should be present and can be recognized and thus provide binding sites for effector proteins (Jenuwein and Allis, 2001).

### Regulation of Post-Translational Modifications of Histones

Three main classes of enzymes involved in the PTMs of histones can be distinguished according to their function: “writers”, “readers” and “erasers”. Writers are enzymes that catalyze the addition of methyl, acetyl or other chemical groups to histone tails, for example Polycomb Repressive Complex 2 (PRC2), SUV39H, DOT1L, with histone methyltransferase (HMT) activity. Readers are proteins, that are able to recognize and bind to specific modifications, for example, PRC1, which reads methylation of H3K27 caused by PRC2 and is subsequently involved in histone ubiquitinization, or chromodomain helicase DNA binding protein 1 (CHD1). Erasers are enzymes such as histone deacetylases (HDACs), or lysine specific demethylase (LSD; KDM6B), which are involved in the removal of modifications (Goldberg et al., 2007; Weng et al., 2012). Each modification may have several writers, readers and erasers (Kouzarides, 2007).

**Figure 3:**
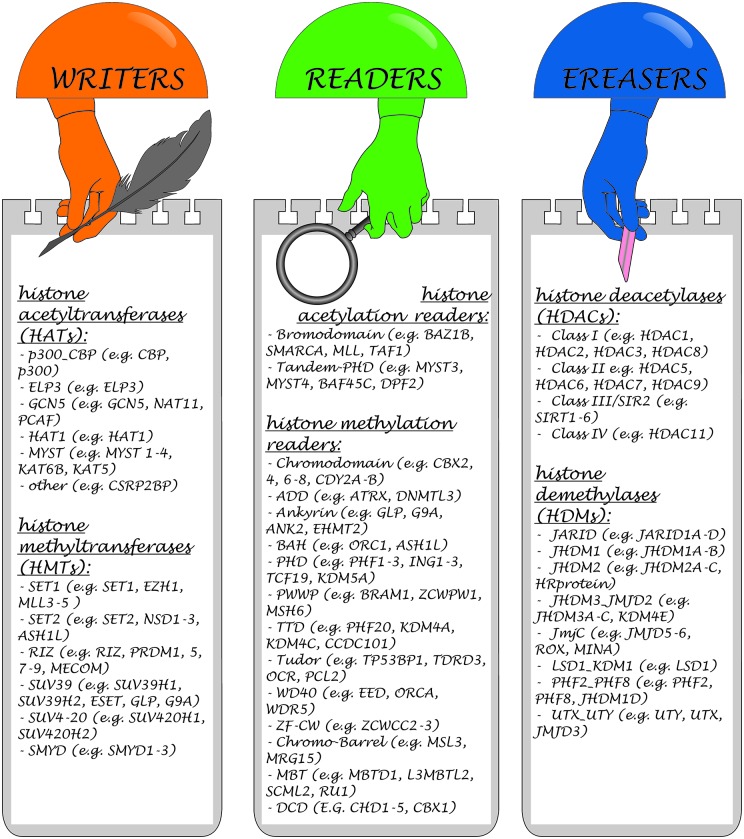
**Classification of enzymes involved in the histone PTMs and representative members of each class**.

Over 580 histone regulators from eight model organisms classified into distinct families have been collected in a large database^1^ (Xu et al., 2017). Examples of some compounds belonging to these three groups are presented in Figure 3.

### Histone Acetylation

Histone acetylation is the most well-defined histone modification and is linked with the lysine residues on the N-terminal tails of histones H3 (Lys^9^, Lys^14^, Lys^18^, Lys^23^) and H4 (Lys^5^, Lys^8^, Lys^12^, Lys^16^). During the acetylation process, acetyl groups from Acetyl-Coenzyme A are transferred to the lysine residues of the histone N termini. This changes the charge and decreases the electrostatic interactions between the positively charged histones and the negatively charged DNA. Furthermore, acetylation of key lysine residues of the core histones H3 and H4 inhibits the association of linker histone—H1, which promotes further chromatin decondensation. This process makes the chromatin available to transcription factors through its relaxation (Ridsdale et al., 1990; Lilja et al., 2012). Histone deacetylation, on the other hand, causes chromatin condensation, preventing binding of transcription factors to their target sequences in gene promoters of NSCs resulting in gene suppression (Hsieh and Gage, 2004).

Potentially active euchromatin and inactive heterochromatin exhibit different acetylation patterns. Euchromatin is normally acetylated at lysine 5, 8 12 and 16 on histone H4, whereas in heterochromatin these sites are hypoacetylated (O’Neill and Turner, 1995). The level of histone acetylation is a dynamic process and depends on two groups of enzymes; histone acetyltransferases (HAT), which are capable of activating acetylation, and HDACs removing this modification, i.e., HDAC activity. So far, eighteen HDACs have been identified in mammals. Based on domain organization and sequence homology with yeast, HDACs can be grouped into four classes (Table 1). It has been shown that each class has a different expression pattern. For example, class I HDACs 1, 2, 3, 8 are the most commonly localized in the nucleus of all cells. On the other hand, HDAC4, 5, 7 and 9 (class II) are found in both the nucleus and cytoplasm and exhibit more tissue-specific expression, suggesting that they are involved in cell differentiation and tissue specification processes (Haigis and Sinclair, 2010; Lian et al., 2012; New et al., 2012).

**Table 1:** **Histone deacetylases classification**.

Class		Members	Localization	Description
Class I		HDAC1 HDAC2 HDAC3 HDAC8	nucleus nucleus nucleus primarily nucleus/cytoplasm	– most closely related to the yeast (*Saccharomyces cerevisiae*) transcriptional regulator Rpd3; – they have Zn^2+^ dependent catalytic domain; – contains the arginase-like amidino hydrolases; – ubiquitously expressed in most cell types; – about 370–500 amino acids; – HDAC1-3 possess low enzymatic activities when in isolation; – they may exist in multi-protein repressor complexes in the nucleus which increase their activity, for instance: SIN3/HDAC corepressor complex, NuRD, CoREST, NODE, SHIP1; – HDAC8 is not related with any multi-protein complex, involved in transcriptional repression; – HDACs of these class are very important in regulation of proliferation and in DNA damage response;
Class II	II A II B	HDAC4 HDAC5 HDAC7 HDAC9 HDAC6 HDAC10	nucleus/cytoplasm nucleus/cytoplasm nucleus/cytoplasm nucleus/cytoplasm primarily cytoplasm primarily cytoplasm	– class II is divided into two subclasses: IIA and IIB; – most closely related to the yeast (*Saccharomyces cerevisiae*) deacetylase Hda1; – they have Zn^2+^ dependent catalytic domain; – contains the arginase-like amidino hydrolases; – expression restricted to specific tissues; – about 1000 amino acids; – class II alone have little or no deacetylase activity; – may exist in large protein complexes as, for instance: HDAC–MEF2 complex, N-CoR, SMRT and BCoR; – catalytic domains is located in the COOH terminus of the peptide (exception: HDAC6—contains an internal duplication of two deacetylase catalytic domains); – HDAC7 possesses three repression domains; – HDAC9–many isoforms;
Class III/SIR2		SIRT1 SIRT2 SIRT3 SIRT4 SIRT5 SIRT6 SIRT7	nucleus/cytoplasm nucleus/cytoplasm nucleus/mitochondria mitochondria mitochondria nucleus nucleolus	– belongs to deoxyhypusine synthase (DHS)-like NAD/FAD-binding domain superfamily; – related to the yeast silencing protein Sir2 (Silent information regulator 2); – SIRT1–6 dependent on NAD^+^ for enzymatic activity; – SIRT7 contains putative enzymatic domain; – from 310 (SIRT4) to 747 (SIRT1) amino acids;
Class IV		HDAC11	nucleus/cytoplasm	– contains conserved residues in the catalytic core regions shared by both class I and II of HDACs; – have Zn^2+^ dependent catalytic domain; – contains the arginase-like amidino hydrolases; – lack of Nuclear Localization Signal (NLS); – 347 amino acids; – expression: kidney, heart, brain, skeletal muscle and testis; – HDAC11 is implicated in immune system regulation via regulation interleukin-10 expression

### HDAC Complexes as the Regulators of Histone Acetylation

In mammals, HDACs from class I and II contain two catalytic domains or form dimers. This suggests that some proteins require two active centers within the HDACs complex (Zhang et al., 2006). So far, several complexes containing HDAC1/HDAC2 have been identified.

One of them, the SIN3/HDAC corepressor complex, is a multiprotein complex implicated in the regulation of key biological processes such as cell cycle regulation, differentiation, protein stabilization and destabilization, transcriptional regulation, cellular senescence or energy metabolism by deacetylation of histones H3 and H4. The SIN3/HDAC corepressor complex is comprised of several proteins, including SIN3 Associated Proteins 30 (SAP30), SAP18, HDAC1, HDAC2, retinoblastoma binding protein 4 (RBAP4), RBAP7, SDS3, MeCP2 and Rb-binding protein (RBP1). The modular structure of the SIN3/HDAC corepressor complex enables different outcomes depending on the associated proteins (Silverstein and Ekwall, 2005; Grzenda et al., 2009; Kadamb et al., 2013). Furthermore, the SIN3/HDAC may also act as a scaffold, enabling access for chromatin remodelers such as histone lysine methylases and demethylases or transcription factors. In this manner, SIN3/HDAC may influence chromatin remodeling processes and transcription regulation (Silverstein and Ekwall, 2005; Grzenda et al., 2009; Kadamb et al., 2013).

Another complex containing HDAC1/HDAC2, the nucleosome remodeling and deacetylase complex (NuRD), can act both as nucleosome remodeler and HDAC (Xue et al., 1998). Additionally, the NuRD complex can play an important role in the regulation of gene transcription, DNA repair or maintenance of genome stability. The NuRD is widely considered to be a transcriptional co-repressor complex, however some studies indicate that it may also have a role in the activation of gene expression (Shimbo et al., 2013). The NuRD complex is formed by several subunits, *inter alia*, chromodomain helicase DNA-binding proteins (CHD3, CHD4, CHD5), histone deacetylases (HDAC1 and HDAC2), histone binding proteins (RBAP48 and RBAP46), MBD2, MBD3 and metastasis associated gene 1, 2 and 3 (MTA1, MTA2, MTA3) proteins (Allen et al., 2013). It has been suggested that heterogeneity of the subunits of this complex and different combinations of proteins in these subunits provide unique functional properties (Bowen et al., 2004).

At least two types of complexes containing histones deacetylases, namely Co-REST and N-COR, have been shown to be involved in the neural commitment and differentiation of stem cells.

CoREST (co-repressor for element-1-silencing transcription factor) complex, regulates neuronal gene expression and is abundantly present in mammalian ESCs, neural progenitors and differentiated non-neuronal cells. Furthermore, CoREST is a platform which recruits different epigenetic factors such as MeCP2, HDAC1/2, LSD1, BHC80 and BRAF35 (Shi et al., 2004; Ballas et al., 2005).

In mammalian genomes there are three genes coding the RCOR protein and which form three types of CoREST complexes: CoREST1, CoREST2 and CoREST3. Recent studies have shown that each of them exhibits different activity and function. CoREST3 mostly resembles the CoREST1, however it has weaker transcriptional repressive activity. CoREST2 exhibits lower HDAC activity and is not inhibited by HDACs inhibitors (e.g., SAHA or TSA) like CoREST1 and CoREST3 complexes (Barrios et al., 2014).

The fourth example of the complex containing HDACs is the nuclear receptor co-repressor (N-CoR)/silencing mediator of retinoid and thyroid hormone receptor (SMRT) complex, comprised of several proteins, including HDAC3, transducin β-like protein 1 (TBL1)/TBL1-related protein 1 (TBLR1) and G-protein pathway suppressor 2 (GPS2). It is involved in the regulation of the differentiation of NSCs and in mouse forebrain development (Jepsen et al., 2007).

### The Relevance of Histone Acetylation for the Neural Commitment and Differentiation of Stem Cells

It has been demonstrated that some of the above-mentioned proteins and complexes play an important role during NSC differentiation and CNS development.

For example, SKI (transcriptional regulator, present in progenitor cells and young neurons) level changes during cell development, while its lack leads to reduced progenitor cell number and direct differentiation into neurons. The impact of SKI on neurogenesis may be associated with its ability to negatively regulate the transforming growth factor β (TGFβ) and bone morphogenetic protein (BMP) signaling pathways by binding to SMAD protein complexes. It is postulated that through its influence on the TGFβ pathway, SKI regulates the balance between proliferation and differentiation of NPC during cortical development.

Moreover, SKI interactions with SIN3/HDAC corepressor complex play an important role during cortical development. In was demonstrated that SKI is not universally expressed in whole brain but is present only in proliferating progenitor cells in the ventricular zone and in subtypes of differentiated projection neurons of the cortical plate (Luo, 2003; Baranek and Atanasoski, 2012; Baranek et al., 2012).

It was also shown that MBD3 is an important player during embryonic development. *Mbd3^−/−^* ESCs remain in the pluripotent state and are not able to commit to developmental lineages (Kaji et al., 2006). Furthermore, NuRD can block reprogramming of somatic cells into pluripotent stem cells (Luo, 2003), however, in the presence of NANOG, MBD3/NuRD induces NSC pluripotency (dos Santos et al., 2014). Such results indicate a context-dependent manner of MBD3/NuRD role in facilitating pluripotency and stemness.

CoREST2 complex has been shown to regulate ESC proliferation and pluripotency (Yang et al., 2011), while CoREST1 complex plays a significant role during neuronal differentiation. All CoREST complexes are expressed in the adult rat brain, both in neuronal and glial cells, however during neuronal differentiation CoREST1 and CoREST2 are downregulated, while CoREST3 expression remains unchanged (Sáez et al., 2015).

It has been shown that CoREST complex stops further differentiation of neuronal progenitors by repressing neuronal differentiation genes. The CoREST complex, which has HDAC activity, interacts with an HMT and this leads to H3K4 methylation. The simultaneous histone deacetylation and H3K4 methylation keep neuronal differentiation genes in a poised state, allowing their further activation. Moreover, in adult not-neuronal cells, CoREST complex in cooperation with an H3K4-specific histone LSD1 and H3K9-specific HMT leads to stable inhibition of neural gene expression (Shi et al., 2004; Ballas et al., 2005). Interestingly, it has been shown that CoREST apart from its key role in neuronal differentiation also influences glial subtype specification and oligodendrocyte lineage maturation. This is attributed to the influence of CoREST complex on the expression of glial genes, thus suggesting that CoREST may be involved in the regulation of astrocyte and oligodendrocyte function, including modulation of synaptic plasticity and axonal myelination (Abrajano et al., 2009). Rodenas-Ruano et al. (2012) demonstrated that in rat hippocampal neurons, expression of genes that encode proteins which build subunits of NMDA receptors is also regulated by REST-dependent DNA methylation. On this basis, these authors suggest that REST is an important player involved in experience-dependent activation of genes involved in synaptic plasticity (Rodenas-Ruano et al., 2012). It has also been shown that one of the phenotypes of Huntington’s Disease is correlated with transcriptional misregulations and abnormal cellular distributions of REST in neurons (Zuccato et al., 2007).

The CoREST complex together with the LSD1, and the HDAC1/2 are the main components of the LSD1-CoREST-HDAC (LCH) transcriptional repressor complexes. Recent studies revealed that in mammals there are four forms of LCH, which is associated with alternative splicing isoforms of LSD1 (LSD1, LSD1-2A, LSD1-8A, LSD1-2A/8A). The LSD1-8A and LSD1-2A/8A isotypes have been associated with neuronal differentiation. Moreover, it was shown that different isoforms are expressed during different stages of neuronal development. At the beginning of the neuronal differentiation of rat cortical neurons all LSD1 isoforms are expressed, however the most abundant ones are LSD1-2A and LSD. In the subsequent days of differentiation, increasing expression of LSD1-8A was observed. It was also observed that early-stage silencing of the LSD1-8A isoform causes a delay in neurite maturation, which indicates a role in the regulation of the proper timing of neurite maturation (Zibetti et al., 2010).

Furthermore, histone acetylation plays an important role in both maintenance of ESC pluripotency and neural differentiation. The histone acetylation level is mostly regulated by distinct HDAC members, which are expressed in specific patterns at different neurodevelopmental stages. Qiao et al. (2015) discovered that H3K9Ac occurs in a very specific manner during hESC neural differentiation *in vitro*. They demonstrated that during the first 4 days of hESC differentiation, the H3K9Ac level decreased. This is related to reduced acetylation at several pluripotency genes. Shortly afterwards, in day 4–8, H3K9Ac levels increased along with neural differentiation. Furthermore, they showed that the use of HDAC inhibitors (HDACi) during the first 4 days of differentiation promoted pluripotency and inhibited neural commitment (Qiao et al., 2015).

As previously mentioned, PTMs include histone acetylation, modulate chromatin accessibility and gene expression. Histone acetylation affects recruitment of chromatin modifier enzymes, such as bromodomain-containing proteins (BRD). It was shown that BRDs play a role in ESC self-renewal and development. For example, both BRD2 and BRD4 mutants are embryonically lethal. BRD2 null mice embryos exhibit abnormalities in neural tube development (Shang et al., 2009). Horne et al. (2015) demonstrated that BRD4 binds at the *NANOG* promoter and maintains its expression and is thus critical for the maintenance of ESC pluripotency.

Different HDACs are expressed at multiple developmental stages. For example, HDAC1 is highly expressed in mice NSCs and glia, while HDAC2 expression is present in neural progenitors and in post-mitotic neurons. Moreover, HDAC2 expression is absent in most of fully differentiated glia, suggesting that sequential expression of specific HDACs is necessary for the differentiation of neuronal and glial subtypes (MacDonald and Roskams, 2008). Recent studies suggest that HDAC inhibition influences neurogenesis in the mouse DG by disturbing the ratio of the neural progenitor types (Foti et al., 2013). Similarly, HDAC interferes with self-renewal in the SVZ (Zhou et al., 2011; Foti et al., 2013).

### Histone Methylation and its Regulation

Histone methylation is the process whereby methyl groups are transferred to lysine (K) or arginine (R) residues. Histone methylation is a reversible process and it is catalyzed by either HMTs or histone demethylases (HDMs). There are two major types of HMTs; arginine-specific, catalyzing methylation of arginine residue on histone H3 and H4, and lysine-specific. HMTs, which catalyze lysine methylation, are subdivided into SET domain-containing (e.g., SUV39H1, SUV39H2) and non-SET domain-containing (e.g., DOT1L; Rea et al., 2000; Feng et al., 2002).

There are currently two known groups of histone LSDs: a flavin adenine dinucleotide (FAD)-dependent amine oxidase, and a Fe(II) and α-ketoglutarate-dependent dioxygenase. The first group is represented by LSD1 (Lysine-specific demethylase 1) and the second by the Jumonji domain-containing proteins (JmjC), which are classified into seven subgroups (e.g., lysine-specific HDM 5B, KDM5B; Shi et al., 2004; Tsukada et al., 2006; Li et al., 2014).

Histone methylation may have different outcomes depending on site (H3 K: 4, 9, 27, 36, 79; H4 K30 or R3) and type of methylation (mono-, di-, or tri-methylation—me1, me2 or me3). Methylation of lysine at sites 4, 36 and 79 on histone 3 (H3K4, H3K36, H3K79) correlates with transcriptional activity and is most commonly linked to Trithorax Group (TrxG) proteins, whereas methylation of lysine 9 and 27 on the same histone (H3K9, H3K27) as well as lysine 20 on histone 4 (H4K20) is typically associated with gene repression and Polycomb Group (PcG) proteins (Bernstein et al., 2006; Schuettengruber et al., 2007).

For example, in housekeeping genes of ESCs there are marks characteristic for transcription initiation, H3K4me3, while at developmental genes there are marks typical for active (H3K4me3) and repressive (H3K27me3) genes, while such double modified genes are generally silent (Bernstein et al., 2006). The specific state of the chromatin, which presents marks of activation and inactivation, is described as the “bivalent” state (“poised state”; Boheler, 2009).

In vertebrates, the PcG proteins comprise a class of transcriptional repressors that are found in multi-subunit complexes. There are two types of Polycomb repressive complexes: PRC1, which is involved in the maintenance of gene repression, and PRC2, which is associated with initiation of gene repression (Rajasekhar and Begemann, 2007). Both complexes catalyze histone covalent modifications and dynamically influence cell lineage commitment (Schuettengruber and Cavalli, 2009).

Regulation of developmental activity and pluripotency genes is linked with activity of the PcG, which binds to the methylated lysine in position 27 on histone 3 and silencing genes (Scheper and Copray, 2009). Maherali et al. (2007) suggested that changes in inactivation marks and PcG play a more significant role for the reprogramming process than methylation on lysine 4 of histone 3. Interestingly, during cell differentiation repressive marks (H3K9me3 and H3K27me3), which in ESCs cover around 4% of the genes, expand to 12%–16% (Hawkins et al., 2010).

### The Relevance of Histone Methylation to the Neural Commitment and Differentiation of Stem Cells

During CNS development, the differentiation of each type of progenitor populations is dependent on the activity of various extracellular signals which facilitate access of transcription factors to promoters of specific glial or neuronal differentiation genes. During neural progenitor differentiation into astrocytes, H3K9 methylation of the *GFAP* promoter is replaced by H3K4 methylation. It was demonstrated that during this process the fibroblast growth factor 2 (FGF2) causes inhibition of H3K9 methylation and induces H3K4 methylation, which enables binding of STAT/CBP complex to the STAT binding site at the promoter of *GFAP* and results in gene activation (Song and Ghosh, 2004). Furthermore, recent studies suggest that histone lysine methylation levels are abnormally regulated in the aged hippocampus, as the baseline resting levels for tri-methylation of H3K4me3 and acetylation of H3K9, K14Ac were found to be different in aged rats with memory deficits compared to those of young adults and failed to increase following object-learning activity. However, environmental enrichment promoted object learning in the aged animals suggesting that histone lysine methylation is implicated in the epigenetic benefits of the environment on memory function (Morse et al., 2015).

The EZH2 (enhancer of Zeste homolog 2)—HMT, which is a part of PRC2, represses gene transcription by triple methylation of lysine 27 (H3K27me3). The PRCs have been widely studied in mouse ESCs showing that these complexes play an important role in maintaining pluripotency and regulating cell fate and identity (de Napoles et al., 2004; Leeb and Wutz, 2007; Pereira C. F. et al., 2010). Furthermore, growing evidence suggests that the PRCs also play an important role in different aspects of mammalian development, including the regulation of tissue specific stem and progenitor cells (Leeb and Wutz, 2010; Laugesen and Helin, 2014).

It has been reported that EZH2, PcG protein, the catalytic subunit of its PRC2 complex, keeps a balance between cortical progenitor cell self-renewal and differentiation and affects the course of neurogenesis (Pereira J. D. et al., 2010). During NSC differentiation into neurons, expression of EZH2 decreases, while it is absent when NSC differentiate into astrocytes and remains at a high level during NSC commitment towards the oligodendrocytic lineage (Sher et al., 2008). EZH2 was shown to negatively regulate neuronal induction during mesenchymal stem cell differentiation by binding to the promoter region and suppressing expression of PIP5K1C (protein present in synapses; Yu et al., 2011). Conversely, EZH2 is involved in suppression of GFAP activation, by binding to its promotor site. This is mediated by CHD4 acting as a EZH2 partner, where the dissociation of CHD4 from the PcG is crucial for the onset of gliogenesis (Sparmann et al., 2013). This suggests a specific role of EHZ2/CHD4 interaction in PcG repressive complex in preventing the premature onset of gliogenesis in maintaining the proper developmental balance in different glial populations.

Furthermore, recent studies have shown that during early mouse development redistribution of H3K27me3 conducted by EZH2 takes place and that it coincides with genome-wide accumulation of H3K9me2, which is mediated by the G9A enzyme. This histone H3 lysine 9 dimethylation was found to be crucial for proper development in post-implantation stage as it regulates the expression of genes involved in proliferation and cell fate decisions, such as *Cd3e, Cdkn1a, Asz1, Rhox5, Pou5f1*. Embryos that lacked G9A, which adds methyl groups at specific developmentally linked genes and causes their silencing, exhibited prematurely activated and inappropriate development. Moreover, G9A enzyme was found to be important in the mechanisms responsible for turning off gene expression enhancers (Zylicz et al., 2015).

Sex-determining region Y-related HMG box 2 (SOX 2) is an NSC and NPC marker important for their self-renewal and adult neurogenesis (Graham et al., 2003; Favaro et al., 2009). SOX2 was shown to be actively involved in epigenetic regulation of neural development by binding to the promotors of genes involved in neural differentiation, thus limiting EZH2 binding and preventing PRC2 activity. It was also shown that SOX2 is involved in regulating the expression of genes important during neuronal development, such as neurogenin 2 (*Ngn2*), neurogenic differentiation 1 (*NeuroD1*), brain-derived neurotrophic factor (*Bdnf*) and growth arrest-DNA-damage-inducible beta (*Gadd45b*). This suggests that SOX2 promotes an open chromatin state, thus enabling proneural and early neurogenic gene activation (Amador-Arjona et al., 2015).

Recent evidence suggests that another chromatin component, Zuotin-related factor 1 (ZRF1), may change developmental gene expression by displacing the PRC1 complex from chromatin and is thus involved in cell fate decisions. Moreover, ZRF1 plays a role in transcriptional regulation and cooperates with deubiquitinase enzyme (Richly et al., 2010). Taken together, these studies demonstrate the importance of histone methylation status and appropriate level of enzymes for the proper development, function and maintenance of the nervous system.

## Interaction Between Histone Modifications and DNA Methylation in NSC Commitment and Differentiation

Different histone modifications may interact with DNA methylation and alter the expression of individual genes. Laurent et al. (2010) suggested that the level of DNA methylation is related to local chromatin conformation. Their findings indicated that there is a strong negative correlation between the level of DNA methylation and histone marks of active transcription (H3K4me3). Interaction between DNA methylation and histone modification is associated with the occurrence of one of two families of methylated CpG DNA binding proteins (MBD or BTB/POZ family; Rose and Klose, 2014).

The DNA methylation program as well as the methylation of histone codes are implicated in neural tube closure during embryonic development (Zhou, 2012).The link of this process to the neuronal differentiation was documented by the appearance of expression of neuronal marks such as NESTIN, CRABP (Cellular retinoic acid binding protein), NEU-N (Neuronal Nuclei) and MAP2 (microtubule-associated protein2).

Furthermore, acetylation of histones, together with the methylation status of DNA play an important role in the regulation of differentiation of developmental processes by regulation of tissue-specific genes. Both types of promoters (high and low-CpG-density promoters) in ESCs are rich in regions of unmethylated CpGs. However, when ESCs were differentiated into NPCs, HCP loss of the H3K4me3 mark caused an increase in the DNA methylation level. Meissner et al. (2008) discovered that in ESCs HCP of housekeeping genes are associated with transcription initiation mark on histone 3 (H3K4me3) and as a consequence, they become highly expressed. In contrast, HCP of developmental genes are marked by both H3K4me3 and H3K27me3 (repressive mark), which results in their silencing. Moreover, promoters of housekeeping genes as well as those of developmental genes are tagged with the H3K4me2 mark, which provides an open chromatin conformation.

## Conclusions

The epigenetic regulatory mechanisms of DNA methylation and histone modification are widely involved in reprogramming, maintaining stemness and NSC differentiation. Epigenome modifications accompany developmental processes from the stage of the first embryonic division through stem cell commitment into defined cell subtypes and determination of their topographic distribution in the adult brain. Furthermore, these mechanisms are implicated in basic cellular processes, such as transcription regulation, DNA replication, cell cycle regulation and DNA repair. Their failure may thus result in severe neurodevelopmental pathologies as well as neurodegenerative diseases. Loss of specific enzymes responsible for deacetylation is associated with embryo lethality, which confirms their importance in embryonic development. Methylation and acetylation are further implicated in cognitive processes such as learning/plasticity and memory formation. The elucidation of these mechanisms in the context of epigenetic modifications may therefore be instrumental for our ability to understand and influence damage and repair processes in the developing as well as adult CNS.

## Author Contributions

LB and MP: substantial contributions to the conception or design of the work; drafting the work and revising the work critically for important intellectual content; final approval of the version to be published; agreement to be accountable for all aspects of the work. IS-G: substantial contributions to the conception or design of the work; drafting the work; final approval of the version to be published; agreement to be accountable for all aspects of the work. JA, IS and AS: substantial contributions to the conception or design of the work; revising the work critically for important intellectual content; final approval of the version to be published; agreement to be accountable for all aspects of the work.

## Conflict of Interest Statement

The authors declare that the research was conducted in the absence of any commercial or financial relationships that could be construed as a potential conflict of interest. The reviewer NNK and handling Editor declared their shared affiliation, and the handling Editor states that the process nevertheless met the standards of a fair and objective review.

## Footnotes

^1^http://weram.biocuckoo.org/
